# Clinical Significance of the Inferomedial Orbital Strut in Orbital Blowout Fractures: Incidence of Symptomatic Diplopia in a Fractured vs. Intact Strut

**DOI:** 10.3390/jcm13133682

**Published:** 2024-06-24

**Authors:** Steffani Krista Someda, Hidetaka Miyazaki, Hirohiko Kakizaki, Yasuhiro Takahashi

**Affiliations:** Department of Oculoplastic, Orbital & Lacrimal Surgery, Aichi Medical University Hospital, Nagakute 480-1195, Aichi, Japan; steffsomeda@gmail.com (S.K.S.); miyaosur@gmail.com (H.M.); cosme_geka@yahoo.co.jp (H.K.)

**Keywords:** blowout fracture, diplopia, inferomedial orbital strut, medial orbital wall fracture, orbital floor fracture

## Abstract

**Background/Objectives**: This study aims to compare the clinical findings, particularly symptomatic diplopia, associated with an inferomedial orbital strut fracture versus intact strut and to determine the clinical significance of the inferomedial orbital strut in patients with orbital floor and medial orbital wall fractures. **Methods**: A 10-year retrospective observational study involving orbital blowout fracture cases was conducted in our institution. Patients with fractures of the orbital floor medial to the infraorbital groove and medial orbital wall, as seen on computed tomography (CT) scans, were included in this study. Patients with concomitant orbital rim fracture and those with old orbital fractures were excluded. Fracture of the inferomedial orbital strut was diagnosed via coronal CT images and patients were classified into those with an inferomedial orbital strut fracture and those without. **Results**: A total of 231 orbits from 230 patients was included in the study (fractured strut on 78 sides and intact strut on 153 sides). Approximately 2/3 of patients in both groups had the field of binocular single vision in primary position upon first examination (*p* = 0.717). Patients with strut fractures demonstrated only comminuted or open fractures, while those without strut fractures showed diverse fracture patterns (*p* < 0.001). **Conclusions**: Inferomedial orbital strut fracture does not automatically result in diplopia in patients with orbital blowout fractures. The integrity of the orbital periosteum plays a more essential role in hampering extraocular muscle displacement, thereby preventing symptomatic diplopia in these patients.

## 1. Introduction

In 1992, Goldberg et al. introduced the anatomical concept of the inferomedial orbital strut, which was utilized during transconjunctival orbital decompression in patients with dysthyroid optic neuropathy in order to prevent ocular dystopia [1]. This study was conducted based on the findings of Long and Baylis who documented patients having marked postoperative hypoglobus following inferomedial orbital decompression surgery [2]. In 1999, Burm et al. also documented the presence of a “bony buttress” demarcating the medial and inferior orbital walls, further implicating its importance in supporting these orbital walls and to prevent diplopia caused by globe displacement [3]. And in the year 2000, Kim et al. published a more comprehensive study on this inferomedial orbital bone structure [4]. This strut of bone, measuring 5 to 7 mm at its widest anterior portion, was found to be anchored firmly at the orbital rim and supported by the medial wall of the maxillary antrum [1,4]. Apart from their finding that ocular dystopia was prevented when the strut was utilized and left intact, this study concluded that the bony inferomedial structure can serve as a medial supporting “ledge” for orbital floor reconstruction [1,4], and this concept holds actual truth. Reconstruction of the medial and inferior orbital walls, in fact, uses the inferomedial strut as an important landmark in positioning the orbital implant of choice [5]. The fracture of this bony strut can, therefore, cause impending trouble for orbital wall reconstruction [5,6,7]. Furthermore, previous studies on orbital decompression have reported an increased incidence of postoperative diplopia, or non-resolution of pre-existing diplopia, in patients whose inferomedial orbital struts were surgically removed [1,8,9,10]. The question now is whether the risk of clinically significant diplopia also increases in orbital blowout fracture patients with concomitant strut fractures.

A spontaneous clinical improvement in patients conservatively treated for orbital blowout fractures has been documented in the literature [11,12]. However, the significance of the inferomedial orbital strut in preventing fracture-related symptomatic diplopia has yet to be established. This study, therefore, aims to determine if a fracture of the inferomedial orbital strut would result in clinically significant diplopia, based on binocular single-vision (BSV) testing, in patients with inferior and medial orbital wall fractures, as well as to determine the type of fractures associated with an inferomedial orbital strut fracture.

## 2. Materials and Methods

This was a retrospective, observational study including all patients with orbital fractures who were referred to our service from May 2013 to April 2023. Our hospital introduced an electronic medical chart system in May 2013. Patients with fractures of the orbital floor medial to the infraorbital groove and medial orbital wall were included in this study. Patients with a concomitant orbital rim fracture, i.e., impure orbital fracture, and those with old orbital fractures were excluded from this study.

The data on age, sex, affected side, the time of examination, causes of injury, concomitant ocular/periocular injuries, presence or absence of infraorbital nerve hypoesthesia, fields of BSV examined on the first visit, and surgery were collected. Causes of injury were classified as follows according to our previous study [13]: sports, assault, fall, traffic accident, works, and others. The results of the field of BSV were classified into 5 categories (B1 to B5), according to our previous study [13], as follows: B1, within normal range (±2 × standard deviation); B2, the field of BSV reaches at least 20 degrees superiorly, 40 degrees inferiorly, and 30 degrees horizontally; B3, a smaller field of BSV than B2 but includes primary gaze; B4, the field of BSV does not include primary gaze; and B5, cannot obtain the field of BSV in any direction of gaze. Data on the presence of enophthalmos were not collected because orbital soft tissue edema caused by trauma prevents accurate Hertel exophthalmometric measurements. Data on postoperative findings were not collected due to several reasons: (1) not all patients included in the study underwent surgical reduction; (2) surgeries were performed by different surgeons; (3) there were different follow-up periods among the patients; and (4) some patients were lost to follow-up after the surgery.

Indications for the surgical repair of orbital fractures in our department were determined based on patient age, the field of BSV, and risk of enophthalmos. Surgical reduction was strongly recommended to patients presenting with a field of BSV at B3 or worse, as well as to patients with a large medial orbital wall fracture susceptible to enophthalmos. Surgery was also recommended to young patients, even with the grade of B2, due to their relatively wider range of activity, which required a wider field of BSV, whereas elderly patients had a narrower range of activity and a higher risk of iatrogenic ophthalmoplegia after surgery. Hence, conservative management was more advisable for elderly patients.

**Figure 1:**
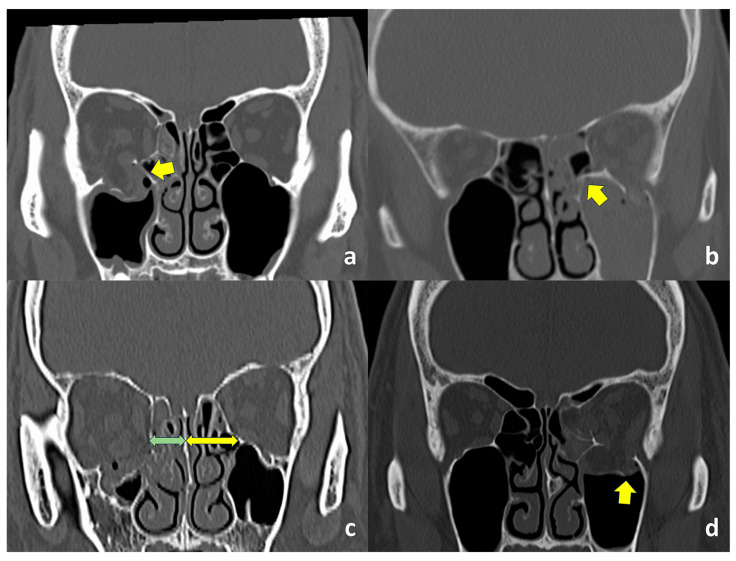
Computed tomographic (CT) findings. (**a**). The inferomedial orbital strut is not fractured (arrow). (**b**). The inferomedial orbital strut is fractured (arrow). (**c**). The distance from the junction between the orbital floor and medial orbital wall and nasal septum is shorter on the affected side (green arrow), compared to the unaffected side (yellow arrow). (**d**). The orbital floor lateral to the infraorbital groove (arrow) is fractured.

Axial and coronal CT images with bone and soft tissue window algorithms were obtained from all patients. Inferomedial strut fractures were diagnosed in cases with apparent strut fractures shown on coronal CT images (Figure 1a,b) or when the distance from the junction between the orbital floor and medial orbital wall and nasal septum was apparently shorter on the affected side (Figure 1c). Orbital fracture patterns, sites, entrapped orbital soft tissues in cases with trapdoor fractures, and concomitant nasal bone fractures were examined. Fracture patterns were classified into comminuted/open, hinged, trapdoor, and linear fractures [14]. The presence or absence of fractures of the orbital floor lateral to the infraorbital groove (Figure 1d) was checked [15]. Entrapped orbital soft tissues in cases with a trapdoor orbital fracture included the extraocular muscles and orbital fat.

Patient age was expressed as means ± standard deviations. Patients were classified into those with and without inferomedial orbital strut fractures. Patient age was compared between the groups using the Student’s *t*-test. A chi-squared test was employed to compare the categorical variables between the groups. All statistical analyses were performed using SPSS™ version 26 software (IBM Japan, Tokyo, Japan). Two-tailed *p* values < 0.05 were deemed to indicate statistical significance.

## 3. Results

Data on patient characteristics and clinical and radiological findings are shown in Table 1, Table 2 and Table 3. Among 1093 sides from 1074 patients with pure orbital fractures, 231 sides from 230 patients with fractures of the orbital floor medial to the infraorbital nerve and medial orbital wall (mean age: 40.8 ± 23.1 years; 160 males and 70 females) were included. All patients were Japanese. The inferomedial orbital strut was fractured on 78 sides in 78 patients, and the strut was intact on 153 sides in 153 patients. One patient with a bilateral orbital floor and medial orbital wall fractures showed an inferomedial orbital strut fracture on one side and no strut fracture on the other side.

Patient age, male-and-female ratio, right-and-left ratio, the time of examination, and ratio of causes of injury were not significantly different between the groups (*p* > 0.050). Concomitant ocular/periocular injuries tended to more frequently occur in patients with strut fractures (26.9% vs. 15.7%; *p* = 0.053). The incidence of infraorbital nerve hypoesthesia was not different between the groups (*p* = 0.449).

Approximately 2/3 patients in both groups had the field of BSV in the primary position (≥B3) on the first examination (*p* = 0.717). A total of 19 patients with “unmeasurable” BSV resulted from either having their vision totally obscured due to the ocular injury or a lack of comprehension in pediatric patients of BSV testing. However, a larger number of patients with strut fractures tended to undergo surgical reductions in orbital fractures, compared to those without strut fractures (70.5% vs. 58.2%; *p* = 0.085).

With regard to the radiological findings, although fracture patterns had variety in patients without strut fractures, patients with strut fractures demonstrated only comminuted/open fractures (*p* < 0.001). According to this finding, all 46 patients with orbital trapdoor fractures did not sustain strut fractures (*p* < 0.001). Although the incidence of concomitant orbital floor fractures lateral to the infraorbital groove was slightly higher in patients with strut fractures (19.2% vs. 13.7%), the difference did not show statistical significance (*p* = 0.338). Concomitant nasal bone fractures were more frequently shown in patients with strut fractures (10.3% vs. 2.6%; *p* = 0.024).

## 4. Discussion

Symptomatic diplopia associated with orbital blowout fractures was first documented in 1957 by Converse and Smith [16]. This usually occurs due to ocular deviation caused by the entrapment of one or more extraocular muscles [16]. The fracture can also lead to the direct injury of the muscle, i.e., laceration, disinsertion, intramuscular hemorrhage, or damage to the nerve controlling eye movement [17]. In fractures involving more than half of the orbital floor, there is also the possibility of the hypoglobus likewise resulting to diplopia [2,16]. Retrospective studies published within the past decade have reported on the incidence of diplopia caused by orbital blowout fractures to be ranging from 20% to as high as 83% [11,12,18]. However, these studies did not indicate the presence of strut fractures. In our previous studies, the incidence of inferomedial strut fractures in patients with orbital blowout fractures was found to be 9% of the sample population (45 out of 475 cases) in one study and 6% (41 out of 671 orbits) in another study [13,19]. Although the incidence rates appear to have insignificant numbers, the implications of these types of fractures will nonetheless aid the clinician in managing such cases.

In our present study, 21 patients with strut fractures (26.9%) were found to have BSV worse than B3, which indicates the presence of diplopia on the primary position of gaze, while 33 patients presenting with similar BSV outcomes had intact inferomedial struts (21.6%). Although the group with fractured struts had a slightly higher frequency of diplopia on primary gaze, statistical analysis showed no significant difference between the fractured strut group and the intact strut group. According to previous studies on medial orbital wall decompression, diplopia occurred when the inferomedial orbital strut was not utilized [4,14]. In contrast, our study has found that strut fractures do not necessarily result in diplopia in orbital blowout fractures. A study by Mansour et al. has similarly found that the involvement of the inferomedial strut is not predictive of the development of diplopia requiring surgical intervention for orbital blowout fractures [20]. Despite having intra-orbital contents herniating through the fracture site, the integrity of the periosteum may actually be more important in preventing clinically significant diplopia [14]. In patients with strut fractures but an intact periosteum, the periosteal layer seems to serve as a hammock that keeps the orbital contents, especially the extraocular muscles, from becoming displaced or incarcerated. This is important for maintaining adequate ocular movement and preventing diplopia, which is mainly caused by the entrapment of extraocular muscles and the intrinsic fibrosis of adjacent fibrofatty tissue secondary to trauma [21].

The presumptive diagnosis of periosteal tear can be made via CT or MRI when the herniated orbital contents do not show a smooth margin [6] or when prolapsed orbital fat crosses over the area of bone fragmentation [21]. However, the presence of a periosteal tear can be quite difficult to ascertain based simply on the radiologic configuration of orbital fat herniation. As such, surgical exploration is still necessary to provide a definitive diagnosis and to address the cause of the diplopia. Although the question of whether muscle impingement can still occur, despite having an intact periosteum, is yet to be investigated, the integrity of the periosteum is definitely much more important than the integrity of the inferomedial orbital strut in the prevention of clinically significant diplopia.

In the fractured strut group, the percentage of patients who underwent surgery was also noted to be higher (70.5%) as compared to the intact strut group (58.2%). Damage to the inferomedial strut could have led to an inadvertent expansion of the orbital space, resulting in clinically significant enophthalmos necessitating surgical intervention [22,23,24]. On the other hand, some of the patients in the intact strut group presented with only fat incarceration and no significant enophthalmos. For this reason, surgery was not indicated.

In the coronal view of orbital imaging, the inferomedial orbital strut can be identified as the bony junction between the medial and inferior orbital walls (Figure 1) [25]. The most common site of fracture, based on our previous study, was found to be the orbital floor medial to the infraorbital nerve [13]. This inferomedial portion of the bony orbit appears to be thinner than the portion of the orbital floor lateral to the infraorbital nerve [14], where the maxillary and zygomatic bones meet, and the integrity of the inferomedial strut becomes even more important to maintain support of the orbital contents. As a buttress of the orbit, the fracture of the inferomedial orbital strut, hence, can be confirmed on imaging when there is a shortening of the distance between this bony junction and the nasal septum (Figure 1c).

Kim et al., in their anatomic study of the inferomedial orbital strut and its clinical implications in globe dystopia after orbital decompression surgery, found that some patients may be prone to concomitant strut fractures due to the presence of pneumatization by the maxillary sinus anteriorly and ethmoid sinus posteriorly [4]. Based on a study by de Silva and Rose, strut fractures were also more common in African (37%) and Asian (30%) patients as compared to the Caucasian patients (13%), probably due to either a greater impact from the trauma or weaker strut associated with these races [26]. Although a recent study by Chan et al. found no significant inter-ethnic variations in the medial orbital wall among Caucasians, the study, nonetheless, found that Chinese, Malays, and Indians had their posterior ethmoidal wall anterior to the posterior maxillary wall, as opposed to Caucasians who had their posterior maxillary wall anterior to the posterior ethmoidal wall [27]. Demographic factors, therefore, contribute to the higher incidence of inferomedial orbital strut fractures resulting from anatomical variations found in certain populations.

The results of the study show that the percentage of patients with ocular injuries is higher in the fractured orbital strut group (26.9%) as compared to the intact-strut group (15.7%). This result is consistent with our previous study showing a high prevalence of inferomedial orbital strut fractures in patients with both pure orbital fractures and lacrimal drainage system injuries [28]. The risk of ocular injuries is lower in patients with orbital blowout fractures, compared to those without fractures, because pressure on the globe at the moment of impact with a material escapes through the fracture site [14,29]. The finding of the present study may likely be due to a high-velocity or a greater force of impact causing both ocular injuries and the strut fractures in these group of patients.

With regards to the concomitant nasal bone fractures found more frequently in patients with strut fractures, this correlation can be explained by the buckling force of impact on the medial orbital rim [3], inevitably causing nasal bone fracture and possibly affecting the most fragile medial portion of the orbital strut.

This study found intact inferomedial orbital struts in 46 patients with orbital trapdoor fractures, although all patients in the fractured strut group showed comminuted or open fractures. A similar finding was reported in the 1999 study by Burm et al. [3]. The likelihood that trapdoor fractures tend to occur in younger patients can explain this phenomenon [14]. Higher bone elasticity found in younger patients decreases the risk of acquiring comminuted fractures [14,30,31], thereby decreasing the risk of concomitant damage to the orbital strut. Despite having all patients with trapdoor fractures in the intact strut group, there was no significant statistical difference in the frequency of symptomatic diplopia between the two study groups. This may be due to the inclusion of only two cases with inferior rectus muscle incarcerations in the intact strut group. Some patients presenting with only orbital fat incarceration do not experience diplopia [32,33]. The main cause of diplopia in cases with orbital fat incarceration is the concomitant incarceration of the inferior oblique muscle branch of the oculomotor nerve, and the incidence of this complication is found to be only 18% [14]. This low incidence may also explain why there is no significant difference in the frequency of symptomatic diplopia between the study groups.

This study has its limitations. For one, the study was performed in a single institution and included consecutive patients diagnosed with orbital blowout fractures. Therefore, randomization of the sample population was not achieved. Furthermore, all patients included were of Japanese descent. Although patient age and sex had no statistically significant difference among the study population, other demographic factors, such as race, can imply possible anatomical differences that may affect the generalizability of the study outcome [27,34]. Lastly, the study was retrospective in nature. The recommendation for future studies would be to conduct a prospective multi-center study that can also factor in populations with different ethnicities. In addition to this, the authors recommend further investigation of the likelihood of diplopia caused by orbital tissue incarceration in the absence of periosteal injury in orbital blowout fracture patients.

## 5. Conclusions

In conclusion, we compared clinical characteristics between patients with and without inferomedial orbital strut fractures. Although fracture patterns and frequency of ocular and periocular injuries tended to be different between the groups, fracture of the inferomedial orbital strut did not affect BSV findings in patients with orbital blowout fractures.

## Figures and Tables

**Table 1:** Data on patient characteristics.

Items	Total	Strut Fracture	No Strut Fracture	*p* Value
Number of patients/sides	230/231	78/78 (33.8%)	153/153 (66.2%)	
Age (years)	40.8 ± 23.1	43.3 ± 22.1	39.6 ± 23.5	0.240
M/F	160 (69.6%)/70 (30.4%)	56 (71.8%)/22 (28.2%)	105 (68.6%)/48 (31.4%)	0.653
R/L	109 (47.2%)/122 (52.8%)	35 (44.9%)/43 (55.1%)	74 (48.4%)/79 (51.6%)	0.677
Time of examination (days)	8.0 ± 5.3	7.7 ± 5.5	8.3 ± 5.0	0.499
Causes of injury				
Sports	69 (29.9%)	19 (24.4%)	50 (32.7%)	0.638
Assault	34 (14.7%)	12 (15.4%)	22 (14.4%)	
Fall	81 (35.1%)	31 (39.7%)	50 (32.7%)	
Traffic accident	25 (10.8%)	10 (12.8%)	15 (9.8%)	
Work	8 (3.5%)	3 (3.8%)	5 (3.3%)	
Others	14 (6.1%)	3 (3.8%)	11 (7.2%)	

M, male; F, female; R, right; L, left.

**Table 2:** Data on clinical findings.

Items	Total	Strut Fracture	No Strut Fracture	*p* Value
Number of patients with concomitant ocular/periocular injuries (some overlapped)	45 (19.6%)	21 (26.9%)	24 (15.7%)	0.053
Hyphema	8	5	3	
Iritis	1	0	1	
Vitreous hemorrhage	2	1	1	
Retinal hemorrhage	1	1	0	
Serous macular detachment	2	0	2	
Maculopathy	1	1	0	
Commotio retinae	13	5	8	
Retinal tear	1	1	0	
Macular hole	3	2	1	
Choroidal rupture	1	1	0	
Globe rupture	3	0	3	
Orbital compartment syndrome	1	0	1	
Traumatic mydriasis	3	1	2	
Eyelid laceration	4	3	1	
Traumatic ptosis	8	5	3	
Floppy eyelid	1	1	0	
Canalicular laceration	1	0	1	
Nasolacrimal canal fracture	2	1	1	
Traumatic superior oblique palsy	1	0	1	
Optic nerve canal fracture	1	1	0	
Infraorbital nerve hypoesthesia	69 (29.9%)	26 (33.3%)	43 (28.1%)	0.449
Field of BSV				
B1	32 (15.2%)	8 (11.3%)	24 (17.1%)	0.717
B2	57 (27.0%)	18 (25.4%)	39 (27.9%)	
B3	68 (32.2%)	24 (33.8%)	44 (31.4%)	
B4	25 (11.8%)	9 (12.7%)	16 (11.4%)	
B5	29 (13.7%)	12 (16.9%)	17 (12.1%)	
Unmeasurable	19 *	7	13	
Number of patients who underwent surgery	144 (62.6%)	55 (70.5%)	89 (58.2%)	0.085

BSV, binocular single vision. * One case had a strut fracture on one side and no strut fracture on the other side.

**Table 3:** Data on radiological findings.

Items	Total	Strut Fracture	No Strut Fracture	*p* Value
Fracture patterns (floor/medial)				
Comminuted/comminuted	129 (55.8%)	78 (100.0%)	51 (33.3%)	<0.001
Hinged/hinged	30 (13.0%)	0	30 (19.6%)	
Trapdoor/trapdoor	18 (7.8%)	0	18 (11.8%)	
Linear/linear	2 (0.9%)	0	2 (1.3%)	
Comminuted/hinged	12 (5.2%)	0	12 (7.8%)	
Comminuted/trapdoor	13 (5.6%)	0	13 (8.5%)	
Comminuted/linear	1 (0.4%)	0	1 (0.7%)	
Hinged/comminuted	5 (2.2%)	0	5 (3.3%)	
Hinged/trapdoor	6 (2.6%)	0	6 (3.9%)	
Hinged/linear	1 (0.4%)	0	1 (0.7%)	
Trapdoor/comminuted	3 (1.3%)	0	3 (2.0%)	
Trapdoor/hinged	4 (1.7%)	0	4 (2.6%)	
Linear/comminuted	4 (1.7%)	0	4 (2.6%)	
Linear/trapdoor	3 (1.3%)	0	3 (2.0%)	
Concomitant orbital floor fracture lateral to infraorbital groove	36 (16.9%)	15 (19.2%)	21 (13.7%)	0.338
Incarcerated tissues				
Inferior rectus muscle	1 (0.4%)	0	1 (0.7%)	<0.001
Inferior rectus muscle (floor) + orbital fat (medial)	1 (0.4%)	0	1 (0.7%)	
Orbital fat (floor and/or medial)	45 (19.5%)	0	45 (29.4%)	
Number of patients with concomitant nasal bone fracture	12 (5.2%)	8 (10.3%)	4 (2.6%)	0.024

## Data Availability

Data supporting the results of this study are available upon request.
